# A functional variant rs4442975 modulating FOXA1-binding affinity does not influence the risk or progression of breast cancer in Chinese Han population

**DOI:** 10.18632/oncotarget.13168

**Published:** 2016-11-07

**Authors:** Yi Zhang, Yan Li, Shaojie Jiang, Wei Chen, Jiao Lou, Juntao Ke, Jiaoyuan Li, Ying Zhu, Yajie Gong, Yang Yang, Jianbo Tian, Xiating Peng, Danyi Zou, Jing Gong, Jiang Chang, Xiaoping Miao, Rong Zhong

**Affiliations:** ^1^ Department of Epidemiology and Biostatistics and State Key Laboratory of Environment Health (Incubation), Ministry of Education Key Laboratory of Environment & Health, Ministry of Environmental Protection Key Laboratory of Environment and Health (Wuhan), School of Public Health, Tongji Medical College, Huazhong University of Science and Technology, Wuhan, Hubei, PR China; ^2^ Department of Preventive Medicine, School of Public Health, Zunyi Medical University, Zunyi, Guizhou, China; ^3^ Department of Gastroenterology, Jing Zhou Central Hospital, Jingzhou, China; ^4^ Zhuhai Center for Chronic Disease Control, Zhuhai, China

**Keywords:** breast cancer, FOXA1, genetic variants, susceptibility, case-control study

## Abstract

The DNA-binding protein FOXA1 has been shown to regulate nearly all estrogen receptor-chromatin interactions, thereby influencing target gene expression levels in breast cancer (BC) cells. Recently, the rs4442975 T-allele, which disrupts the recruitment of FOXA1 and interacts with the *IGFBP5* promoter, was associated to BC susceptibility in a European population. We conducted a hospital-based case-control study that included 1227 cases and 1285 controls to explore the potential association between rs4442975 and BC risk in Chinese Han population, and the effect of this SNP on BC progression was also observed in cases. No significant associations between rs4442975 and BC risk were observed under any genetic models, with an odds ratio of 0.96 (95% confidence interval = 0.81-1.15) under the additive model. When stratified based on estrogen or progesterone receptor expression, smoking or drinking habits, or menopausal status, similar negative associations were observed for all subgroups. No significant associations were observed between rs4442975 and traditional progression factors such as tumor size, nodal status, distant metastasis, or TNM staging. These results reveal that rs4442975 may not confer a risk of BC occurrence or progression in the Chinese Han population, which indicates a distinct association related to genetic heterogeneity across ethnic populations.

## INTRODUCTION

Breast cancer (BC) remains a global public health issue and the most common cancer in females worldwide. An estimated 1.67 million newly diagnosed BC cases and 521,907 deaths occurred in 2012 according to the database of the International Agency for Research on Cancer (IARC) [1], accounting for 25.2% of total cancer cases and 14.7% of cancer deaths among females. In China, the incidence of breast cancer has continuously increased over the past few decades. It was estimated that the morbidity and mortality of BC in Chinese females in 2015 were 26.86/100,000 and 6.95/100,000, respectively [2]. In order to effectively reduce the morbidity and mortality, it is urgent to clarify the mechanism of BC pathogenesis.

Genome-wide association studies (GWASs) have revealed multiple loci and single nucleotide polymorphisms (SNPs) associated with BC risk [3, 4]. The function of the majority of BC risk-associated SNPs, located in non-coding regions, remains unclear, complicating the search for potential mechanisms underlying those associations [5, 6]. Regulatory mechanisms of risk-associated SNPs identified by GWAS have, to date, been gradually discovered. As a consequence, fine mapping of the non-coding variants associated with BC risk has been the main point of systemic post-GWAS functional characterization. Remarkably, BC risk-associated SNPs identified by GWAS were enriched in the binding sites for the transcription factors FOXA1 and estrogen receptor 1 (ESR1) [7], which suggested FOXA1 contributes to BC development by regulatory mechanisms related to estrogen receptor (ER) signaling [8, 9].

FOXA1 is a pioneer factor that directly binds and modulates compacted chromatin [10], which in turn facilitates or inhibits the recruitment of other transcription factors, particularly steroid hormone receptors including ER [11–13]. Direct evidence suggests that FOXA1 regulates almost all estrogen receptor-chromatin interactions, thus influencing target gene expression levels in breast cancer cells [14, 15]. Silencing of FOXA1 leads to the global inhibition of ER binding and transcriptional activity [15, 16]. Previous studies showed that the deregulation of FOXA1 played a potentially critical role in the carcinogenesis of breast cancer by disturbing ER binding [15]. Inactivating mutations of FOXA1 have been observed in breast cancer by exome sequencing study [17]. Additionally, differential ER binding is associated with the prognosis of BC patients [18], and co-recruitment of FOXA1 appears to be related to reprogramming of ER binding. Several reports showed that FOXA1 expression levels significantly correlated with better breast cancer-specific survival [19–24]. Therefore, it has been proposed that genetic variants in FOXA1 binding sites might affect ER binding to FOXA by modulating the affinity of chromatin, thereby regulating target gene expression levels and eventually contributing to the risk of breast cancer initiation and progression.

A recent GWAS study [25] from Ghoussaini M et al. demonstrated that the variant rs4442975, which is strongly correlated with rs13387042 [26, 27] (r^2^>0.8), and disrupts the recruitment of FOXA1 [25, 28], was associated with elevated BC risk in Europeans due to a resulting reduction in *IGFBP5* expression. However, the association between rs4442975 and BC risk in Chinese Han population has yet to be reported.

Given the genetic heterogeneity across populations, cell types, and tissue of breast cancer, we carried out a hospital-based case-control study in a Chinese Han population to explore the role of rs4442975 in susceptibility to BC, and the effect of this SNP on BC progression was also observed in cases.

## RESULTS

### Subject characteristics

The characteristics of study participants are presented in Table 1. The controls and cases were well matched in the distribution of age and menopausal status, with *P*-values of 0.483 (*t=*0.701) and 0.619 (*x^2^*=0.247), respectively. Additionally, there were no differences in drinking or smoking habits between control and case group. Among BC patients, 760 (61.94%) cases were ER positive and 463 (37.73%) were ER negative, 4 (0.33%) cases were ER unknown. 683 (55.66%) and 537 (43.77 %) cases were classified as progesterone receptor (PR) positive and PR negative, respectively. Additionally, there were 615 (50.12%) cases with lymph node metastasis.

**Table 1:** Characteristics of the participants in the case-control study

Variables	Case (1227) No(%)	Control (1285) No(%)	*P*
**Age** (Mean ± SD)	48.91±9.69	48.64±9.50	0.483^a^
**Smoking**			0.581^b^
Yes	9(0.73)	12(0.90)	
No	1218(99.27)	1273(99.10)	
**Alcohol use**			0.759^b^
Yes	20(1.63)	19(1.50)	
No	1207(98.37)	1266(98.50)	
**Menopausal status**			0.619^b^
Premenopausal	690(56.23)	743(57.80)	
Postmenopausal	524(42.71)	542(42.20)	
**Estrogen receptor**			
Positive	760(61.94)		
Negative	463(37.73)		
**Progesterone receptor**			
Positive	683(55.66)		
Negative	537(43.77)		
**Tumor size**			
≤ 5 cm	859(70.01)		
> 5 cm	95(7.74)		
**Lymph node metastasis**			
Yes	615(50.12)		
No	505(41.16)		
**Distant metastasis**			
Yes	96(7.82)		
No	1052(85.74)		
**TNM stages**			
TNM I-II	775(63.16)		
TNM III-IV	304(24.78)		

a P value was calculated by t test

b P value was calculated by x2 test.

### The association between rs4442975 and BC risk

The genotypes of rs4442975 in controls conformed to the Hardy-Weinberg equilibrium (*P* = 0.829), and the minor allele frequency was consistent with the 1000 Genomes Project. The genotype distribution of rs4442975 and its associations with BC risk are presented in Table 2. Compared with individuals carrying the TT genotype, there was no increased risk of BC for individuals with TG and GG genotypes, with an OR of 0.98 (95% CI: 0.81-1.19) after adjusting for drinking, smoking, menopausal status, and age. Meanwhile, no significant association between rs4442975 and BC risk was found under the heterozygote or homozygote models (OR=0.96, 95% CI: 0.78-1.17 and OR=0.97, 95% CI: 0.50-1.88 respectively). A similar negative result was also observed in the additive model.

**Table 2:** The associations between rs4442975 and BC risk in Chinese population

Variables	Case(1227) No. (%)	Control(1285) No. (%)	MAF in Control	MAF in CHB^a^	OR(95%CI); *P^b^*
**rs4442975**	1201(97.88)	1270(98.83)	0.119	0.112	
TT	942(78.43)	985(77.56)			1.00
TG	242(20.15)	266(20.94)			0.96(0.78-1.17); 0.662
GG	17(1.42)	19(1.50)			0.97(0.50-1.88); 0.928
Dominant					0.98(0.81-1.19); 0.812
Additive					0.96(0.81-1.15); 0.678

a MAF was downloaded from 1000 Genome Project data among Han Chinese in Beijing individuals;

b ORs and 95% CIs were calculated by unconditional logistic regression after adjusting for age, smoking, alcohol use, and menopausal status.

Stratified analysis was performed according to ER and PR expression, smoking and drinking habits, and menopausal status. No significant association between rs4442975 and BC risk was found in ER+, ER-, PR+, PR-, premenopausal, postmenopausal, non-smoker, or nondrinker subgroups (Supplementary Table S1, Supplementary Table S2, and Supplementary Table S3).

### The association between rs4442975 and progression of BC

We further evaluated the association between rs4442975 and clinicopathological features such as tumor size, lymph node involvement, distant metastasis, and TNM stage (Table 3), which are typical indicators of BC progression. No associations were found between the SNP and any of the studied prognostic factors under the additive model: tumor size (*P* = 0.823), lymph node involvement (*P* = 0.933), distant metastasis (*P* = 0.154), and advanced TNM stage (*P* = 0.765). A similar negative result was observed in the dominant model.

**Table 3:** Association of rs4442975 genotypes with clinicopathologic parameters

Parameter	Parameter category No. (%)		OR(95%CI); P^a^
**Tumor Sizes**	**≤ 5 cm(859)**	**> 5 cm(95)**	
**rs4442975**	848(98.72)	92(96.84)	
TT	673(79.36)	71(77.17)	1.00
TG	162(19.11)	21(22.83)	1.31(0.77-2.21); 0.320
GG	13(1.53)	0(0)	—
Dominant			1.15(0.68-1.94); 0.601
Additive			1.06(0.66-1.70); 0.823
**Lymph node metastasis**	**Negative(505)**	**Positive(615)**	
**rs4442975**	493(97.62)	608(98.86)	
TT	390(79.11)	471(77.47)	1.00
TG	93(18.86)	130(21.38)	1.14(0.83-1.55); 0.423
GG	10(2.03)	7(1.15)	0.56(0.21-1.50); 0.251
Dominant			1.07(0.80-1.45); 0.642
Additive			1.01(0.77-1.32); 0.933
**Distant metastasis**	**Negative(1052)**	**Positive(96)**	
**rs4442975**	1032(98.10)	92(95.83)	
TT	806(78.10)	76(82.61)	1.00
TG	209(20.25)	16(17.39)	0.78(0.44-1.39); 0.400
GG	17(1.65)	0(0)	—
Dominant			0.69(0.39-1.24); 0.214
Additive			0.68(0.39-1.16); 0.154
**TNM stages**	**I-II(775)**	**III-IV(304)**	
**rs4442975**	758(97.81)	297(97.70)	
TT	601(79.29)	232(78.11)	1.00
TG	143(18.86)	64(21.55)	1.15(0.82-1.62); 0.419
GG	14(1.85)	1(0.34)	0.16(0.02-1.24); 0.080
Dominant			1.08(0.77-1.51); 0.658
Additive			0.95(0.70-1.30); 0.765

a P values were calculated using unconditional logistic regression after adjusting for age, smoking, alcohol use, menopausal status, ER and PR.

## DISCUSSION

In the present study, we conducted a hospital-based case-control study in a Chinese Han population to explore the potential association between the FOXA1-binding functional variant rs4442975 and BC risk. Our results demonstrated that rs4442975 was not associated with the risk of BC incidence or progression among Chinese population, and negative results were also observed in all of the subgroups stratified by ER, PR, smoking, drinking, and menopausal status.

Rs4442975 is located near a putative regulatory element and interacts with the *IGFBP5* promoter. It has been reported that the cancer-protective T allele of rs4442975 creates a stronger interaction with *IGFBP5* than the G allele, resulting in increased *IGFBP5* expression. Additionally, rs4442975 resides in the FOXA1 binding site with the T allele leading to increased FOXA1 binding [25, 28]. FOXA1 is a pioneer factor and plays an important role in BC risk by interrupting the FOXA1-binding ability which then influences the ER binding and transcription of its target genes [29]. However, no association between rs4442975 and risk of BC incidence or progression was found in this Chinese population.

We propose the following explanation for the conflicting results between different ethnic groups. The differential role of rs4442975 in BC risk between European and Chinese women may be partly due to different genetic backgrounds among populations: MAF (G) was 0.112 in CHB versus 0.509 in EUR. Differences in linkage disequilibrium (LD) patterns across populations may result in the heterogeneity of effect size. So, the variant observed to associate with different effect sizes among different populations may not be the actual causal variant, whereas a variant with similar effect sizes can be causal. Rs4442975 was strongly correlated with rs13387042 (r^2^=0.93), which is located in 2q35 and first identified to contribute to both ER positive and ER negative BC susceptibility in an Icelandic GWAS and then confirmed by a large European replication study [26, 27]. Similarly, rs13387042 was located in a gene desert region with unknown biological function. According to the “multiple enhancers variant” hypothesis [30], we cannot exclude situations where another SNP, yet to be detected, may be in the same LD block of rs4442975 and influence FOXA1 binding. Additionally, breast cancer is highly heterogeneous, both at the tissue and cell level, and risk-associated SNPs may act only on one specific cell type.

The study still had some limitations. Selection bias may exist due to the hospital-based case-control study design. The sample size was not very large to detect the modest effect of the SNP on the risk of BC. Unfortunately we do not have 5-years survival rate, so we could not estimate the effect of rs4442975 on the prognosis.

In conclusion, our study indicates that the variant rs4442975 did not confer increased risk of BC incidence or progression in this population of Chinese Han women. Furthermore, our results emphasize the distinct mechanisms behind genetic heterogeneity across populations, cell types and tissue heterogeneity of breast cancer. Although well designed and larger population-based case-control studies are required to confirm the role of this polymorphism in BC risk across different populations, it is critical to clarify whether the significant association between rs4442975 and BC risk in the European population is a result of other genetically linked causal variants.

## MATERIALS AND METHODS

### Subjects for the case-control study

A hospital-based case-control study of 1227 breast cancer patients and 1285 control subjects was performed to comprehensively examine the association between rs4442975 and the risk of BC in the Chinese population. All subjects in this study are female and unrelated ethnic Han Chinese. There were no age or histology restrictions. Patients with newly diagnosed, histopathologically confirmed, and previously untreated (by radiotherapy or chemotherapy) breast cancer were consecutively recruited between June 2010 and June 2014 at Union Hospital of Huazhong University of Science and Technology (HUST) of Wuhan, Central China. Patients with metastasized cancer from other organs or a history of other cancers were not included in the case group. Control subjects were randomly chosen from a pool of cancer-free people who participated in health examination during the same period at the same hospital. Controls were frequency-matched to the cases by age (±5 years). Tumor stage was evaluated according to the 2002 American Joint Committee on Cancer staging system. At recruitment, written informed consent was obtained from each participant, and then personal characteristics and peripheral venous blood sample (2 ml) were collected from each participant. The definitions of smokers and drinkers have been described previously [31, 32]. Briefly, subjects who had smoked less than 1 cigarette per day and < 1 year or never smoked were classified as non-smokers; otherwise, they were defined as smokers. Subjects who drank more than twice a week and continued for at least 1 year were defined as drinkers, while others were considered as nondrinkers. The study was approved by the institutional review boards of Tongji Medical College of Huazhong University of Science and Technology.

### Genotyping

Genomic DNA was isolated from the 2ml peripheral venous blood samples of all participants using Relax Gene Blood DNA System DP319-02 (Tiangen, Beijing, China) in accordance with the manufacturer's recommended protocol. The genetic variant rs4442975 was genotyped by TaqMan SNP Genotyping Assay using the 7900HT Fast Real-Time PCR System (Applied Bio-systems, Foster City, CA). Genotyping was performed without knowledge of case/control status of the subjects. Approximately 5% of the random samples from cases and controls were genotyped twice, and the results were in 100 % concordance. All methods were carried out in accordance with the approved guidelines.

### Statistical analysis

The statistical power to detect the association between BC and the SNP was calculated by Power v3.0 [33–35]. The distribution difference of demographic characteristics was evaluated by the *t*-test or chi-square test between case and control group. The Hardy-Weinberg equilibrium (HWE) for the genotype frequencies was tested by the goodness-of-fit chi-square test in controls. The odds ratios (ORs) and their 95% confidence intervals (CIs) were calculated by unconditional multivariate logistic regression analysis to estimate the association between genotypes and BC risk after adjusted by smoking, drinking, menopause status, and age.

For rs4442975 with minor allele frequencies (MAF) of 0.119 in controls, the statistical power for our sample size to detect an OR of 1.50 is 0.939. All *P* values were two-sided and *P* < 0.05 was considered statistically significant. All statistical analyses were performed using the SPSS software (V18.0).

## SUPPLEMENTARY MATERIALS TABLES


