# Supplementary material for: A functional variant rs4442975 modulating FOXA1-binding affinity does not influence the risk or progression of breast cancer in Chinese Han population
Source: Oncotarget. 2016 Nov 7;7(49):81691–7. doi: 10.18632/oncotarget.13168 (PMC5348423; doi:10.18632/oncotarget.13168)
Supplement: Supplementary file 1 [file oncotarget-07-81691-s001.pdf]

A functional variant rs4442975 modulating FOXA1-binding affinity does not influence the risk or progression of breast cancer in Chinese Han population

SUPPLEMENTARY TABLES

Supplementary Table S1: Stratified analyses of the association between rs4442975 and BC risk in a Chinese population

rs4442975	Case No. (%)	Control No. (%)	OR (95%CI); *P*[a]
**ER+**	749(98.55)	1270(98.83)	
TT	592(79.04)	985(77.56)	1.00
TG	150(20.03)	266(20.94)	0.94(0.75-1.17); 0.573
GG	7(0.93)	19(1.50)	0.61(0.25-1.46); 0.265
Dominant			0.93(0.75-1.16); 0.517
Additive			0.90(0.74-1.11); 0.319
**ER-**	448(96.76)	1270(98.83)	
TT	348(77.68)	985(77.56)	1.00
TG	90(20.09)	266(20.94)	0.97(0.74-1.27); 0.811
GG	10(2.23)	19(1.50)	1.48(0.68-3.21); 0.327
Dominant			1.03(0.79-1.33); 0.847
Additive			1.04(0.82-1.30); 0.769
**PR+**	671(98.24)	1270(98.83)	
TT	532(79.29)	985(77.56)	1.00
TG	131(19.52)	266(20.94)	0.91(0.72-1.15); 0.426
GG	8(1.19)	19(1.50)	0.78(0.34-1.79); 0.553
Dominant			0.93(0.74-1.16); 0.500
Additive			0.90(0.73-1.11); 0.336
**PR-**	523(97.39)	1270(98.83)	
TT	405(77.44)	985(77.56)	1.00
TG	109(20.84)	266(20.94)	1.01(0.78-1.30); 0.954
GG	9(1.72)	19(1.50)	1.14(0.51-2.54); 0.753
Dominant			1.03(0.80-1.31); 0.844
Additive			1.02(0.82-1.27); 0.841
**Pre-menopausal**	673(97.54)	732(98.52)	
TT	517(76.82)	566(77.32)	1.00
TG	147(21.84)	156(21.31)	1.04(0.81-1.35); 0.742
GG	9(1.34)	10(1.37)	0.97(0.39-2.41); 0.951
Dominant			1.07(0.84-1.38); 0.581
Additive			1.03(0.82-1.30); 0.796
**Post-menopausal**	515(98.28)	538(99.26)	
TT	412(80.00)	419(77.88)	1.00
TG	95(18.45)	110(20.45)	0.88(0.65-1.19); 0.407
GG	8(1.55)	9(1.67)	0.91(0.35-2.38); 0.840
Dominant			0.88(0.65-1.19); 0.401
Additive			0.90(0.69-1.17); 0.428

[a]P values were calculated using unconditional logistic regression after adjusting for age, smoking, alcohol use and other covariates.

Supplementary Table S2: Association between rs4442975 and BC risk in non-smokers

Variables	Case (1218) No. (%)	Control (1273) No. (%)	OR (95%CI); *P*[a]
**rs4442975**	1192(97.87)	1258(98.82)	
TT	933(78.27)	977(77.66)	1.00
TG	242(20.30)	262(20.83)	0.98(0.81-1.20); 0.863
GG	17(1.43)	19(1.51)	0.94(0.50-1.83); 0.863
Dominant			0.99(0.82-1.20); 0.957
Additive			0.98(0.82-1.17); 0.819

[a]ORs and 95% CIs were calculated by unconditional logistic regression after adjusting for age, alcohol use and menopause status.

Supplementary Table S3: Association between rs4442975 and BC risk in non-drinkers

Variables	Case (1207) No. (%)	Control (1266) No. (%)	OR (95%CI); *P*[a]
**rs4442975**	1181(97.85)	1251(98.82)	
TT	923(78.15)	972(77.70)	1.00
TG	241(20.41)	261(20.86)	0.99(0.81-1.20); 0.901
GG	17(1.44)	18(1.44)	1.00(0.51-1.95); 1.000
Dominant			1.00(0.83-1.22); 0.973
Additive			0.99(0.83-1.18); 0.916

[a]ORs and 95% CIs were calculated by unconditional logistic regression after adjusting for age, smoking and menopause status.
